# Epigenetic modification and preliminary investigation of the mechanism of the immune evasion of HL-60 cells

**DOI:** 10.3892/mmr.2015.3526

**Published:** 2015-03-20

**Authors:** JIN HONG LIU, YONG MEI BIAN, YI XIE, DAO PEI LU

**Affiliations:** 1 Department of Hematology, The Fifth People’s Hospital of Shanghai, Fudan University, Shanghai 200240, P.R. China; 2 Department of Pediatrics, Minhang District Maternal and Child Health Care Hospital of Shanghai, Shanghai 201102, P.R. China; 3 Department of Hematology, Huashan Hospital, Fudan University, Shanghai 200040, P.R. China

**Keywords:** leukemia, epigenetic modification, immune escape, class II transactivator, major histocompatibility complex class II

## Abstract

The aim of the present study was to explore the effect of epigenetic modification of class II transactivator (CIITA) methylation on histocompatibility complex (MHC) class II expression and the immune evasion of leukemia HL-60 cells. HL-60 cells were treated with various concentrations of 5-aza-2′deoxycytidine (5-Aza-CdR) and 0.5 *μ*mol/l suberoyl-anilide hydroxamic acid (SAHA) for 24 h and then stimulated by interferon γ (IFN-γ) for 48 h. The mRNA levels of MHC class I, II and co-stimulatory molecules were quantified by reverse transcription polymerase chain reaction (RT-PCR). The levels of CIITA protein were determined by western blot analysis, and the CpG island methylation ratios in the CIITA promoter IV (CIITApIV) were analyzed by bisulfite-sequencing PCR (BSP). MHC I as well as the co-stimulatory molecules CD40 and CD80 were significantly increased following treatment with 5-Aza-CdR + SAHA + IFN-γ (epigenetic groups) compared with those in the control group and IFN-γ group (P<0.05). The expression of MHC class II and CIITA was restored and increased in an 5-Aza-CdR concentration-dependent manner in the three epigenetic groups. The results of the BSP assay showed that the methylation rate of CIITApIV CpG sites decreased with the treatment of epigenetic modification and negatively correlated to the 5-Aza-CdR concentration. This demonstrated that the negative expression of CIITA protein was the key reason for the loss of MHC II expression in HL-60 cells. The results of the present study may help to illustrate the mechanism of immune evasion in HL-60 cells.

## Introduction

Leukemia is a malignant blood disorder and serious threat to human health (1). Tumor cells can not be effectively attacked by the immune system in spite of the presence of tumor-specific antigens, which is known as immune evasion. Previous studies have shown that an important mechanism of the immune evasion of tumor cells is the absent or low expression of major histocompatibility complex (MHC) molecules and co-stimulatory molecules (2–5). However, it has rarely been studied whether or not leukemia cells evade the immune system through this mechanism. The MHC is one of the most important genetic systems for preventing pathogen invasion and maintaining the immune system in higher developed animals (6). As one of MHC class II gene transcription factors, regulatory factor X activity is controlled by class II transactivator (CIITA) (7). CIITA regulates MHC class II expression as a transcriptional activator and as a general transcription factor (8). It is the ‘speed factor’ and ‘molecular switch’ of MHC class II, and quantitatively controls MHC class II mRNA expression (9). Four types of CIITA promoter have been identified in humans (10). In certain instances, the silencing and knockdown of CIITA promoter IV (CIITApIV) have been mostly responsible for failure of interferon (IFN)-γ to induce MHC II gene transcription and the partial silencing of MHCII molecules (11,12).

Epigenetic modifications in cells are closely associated with the occurrence of leukemia. It has been reported that epigenetic abnormalities occurred in human cancer cells and may be the key to initiate tumorigenesis (13,14). DNA methylation and histone modification are two main causes of gene mutation (15). To date, fifteen DNA methylation biomarkers for diagnosis and sub-typing of pediatric acute lymphoblastic leukemia (ALL) have been found (16), and DNA methylation was shown to be an indicator of the ALL sub-type as well as clinical outcome (17). The histone deacetylase inhibitor (HDACi) belinostat (PXD101) inhibited cell growth, induced apoptosis and increased the acetylation of histone H3 and H4 in a dose-dependent manner in promyelocytic leukemia HL-60 and NB4 cells; it is under development as an epigenetic drug for anti-leukemia and differentiation therapy (18). In general, histone acetylation on chromatin and DNA demethylation on cytosine-phosphate-guanine (CpG) loci can loosen the chromatin structure, which aids in the binding of transcription factors to gene control regions and promotes gene expression. Conversely, histone deacetylation and methylation inhibit gene expression (19). Previous studies have shown that epigenetic regulation was able to silence or reduce CIITA in cancer cells (20,21). In a variety of MHC class II-negative tumor cells, elevated levels of chromosomal histone deacetylation and CpG site methylation on the CIITA promoter have been detected; however, treatment with HDACi or DNA methyltransferase inhibitors (DNMTi) increased the expression of MHC II, and the underlying mechanism may be the transcriptional activation of the CIITA gene (21).

If treatment with HDACis or DNMTis has similar effects on leukemia cells to those mentioned above, they may be used as highly effective preventives or anti-leukemia agents. In the present study, leukemia HL-60 cells were treated with the HDACi 5-aza-2′-deoxycytidine (5-Aza-CdR) and/or the DNMTi suberoylanilide hydroxamic acid (SAHA) and then stimulated by IFN-γ to explore their effect on CIITA methylation and the resulting MHC class II expression. The results gave clues on the underlying mechanism of the immune evasion of leukemia HL-60 cells.

## Materials and methods

### Cell lines and agents

The human HL-60 cell line was purchased from Shanghai Institute of Biological Sciences, Chinese Academy of Sciences (Shanghai, China). RPMI 1640 culture medium, fetal calf serum, bovine serum albumin (BSA), dimethylsulfoxide (DMSO) and trypsin were purchased from Gibco-BRL (Invitrogen Life Technologies, Carlsbad, CA, USA). 5-Aza-CdR (Sigma Aldrich, St Louis, MO, USA) and SAHA (Cayman Chemical Company, Ann Arbor, MI, USA) were used for epigenetic modification. IFN-γ (PeproTech, Rocky Hill, NJ, USA) was used for promoting the expression of MHC class II molecules. TRIzol reagent was obtained from Invitrogen Life Technologies. RevertAid™ First Strand cDNA kit and short DreamTaq™ Green polymerase chain reaction (PCR) Master Mix (2X) were products of Fermentas (Thermo Fisher Scientific, Burlington, Canada). PMD18-T cloning vector, *Esherichia coli* DH5α, proteinase K and RNase A were purchased from Takara Biotechnology Co., Ltd. (Dalian, China). Rabbit anti-human CIITA-1 antibody (cat. no. A1709) was purchased from Wuhan Sino-US Sciences Co., Ltd (Wuhan, China). β-actin rabbit polyclonal antibody (cat. no. sc-130657) was purchased from Santa Cruz Biotechnologi, Inc. (Dallas, TX, USA). EZ DNA methylation-Direct kit was purchased from Beijing Tianmo Sci & Tech Development Co., Ltd (Beijing, China). DNA molecular size standard was a product of New England Biolabs Inc (Ipswich, MA, USA). A 100-bp DNA marker was purchased from Generay Biotech (Shanghai, China). Protein standard was a product of Sangon Biotech (Shanghai, China). Tween-20, nitrocellulose membrane, Ponceau S solution and ethylene glycol tetraacetic acid (EGTA) were from Amresco (Solon, OH, USA). Polyacrylamide gel, SDS, isopropyl-β-d-thiogalactoside (IPTG), X-gal and Gel Extraction kit were all purchsed from Shanghai Huashun Biotechnology Co., Ltd. (Shanghai, China). Casein tryptone and yeast extract were from Oxiod (Basingstoke, UK). Brilliant blue G 250 and ampicillin were from Sigma Aldrich.

### Cell culture

HL-60 cells were cultured in RPMI 1640 supplemented with 10% fetal calf serum, 100 U/ml penicillin and 100 *μ*g/ml streptomycin (Sigma-Aldrich) in a 5% CO_2_ atmosphere at 37°C. Cells were subcultured every two days.

### Experimental groups

According to the various final concentrations of drugs in the media, cells were divided into five groups: A) control group treated with phosphate-buffered saline (PBS; Sigma-Aldrich) only for 120 h; B) 1,000 U/ml IFN-γ for 48 h; C-E) 5-Aza-CdR (0.1 *μ*M, 1 *μ*M or 10 *μ*M, respectively, for 48 h) followed by SAHA (0.5 *μ*mol/l) for 24 h, then stimulation with IFN-γ (1,000 U/ml) for 48 h. Experimental conditions in each group were repeated five times. Following treatment, growth conditions and morphological changes of cells were observed.

### Reverse-transcription quantitative PCR

Following treatment, cells from all groups were washed in PBS. Total RNA was isolated using TRIzol and identified using an ultraviolet (UV) spectrophotometer (UV2550; Shimadzu, Kyoto, Japan). cDNA was synthesized using the RevertAid™ First Strand cDNA Synthesis kit. Consulting the sequences in GeneBank, primers for MHC II, MHC, CD40, CD80 and were designed using Primer 5.0 software and synthesized by Invitrogen (Shanghai, China). Primer sequences were as follows: MHC II (HLA-DRA) forward, 5′-GAAATGGAAAACCTGTCACCAC-3′; MHC II reverse, 5′-AAACTCCCAGTGCTTGAGAAGA-3′; MHC I (HLA-A) forward, 5′-GTATTTCTTCACATCCGTGTCC-3′; MHC I reverse, 5′-TTCACATTCCGTGTCTCCTG-3′; CD40 forward, 5′-ACCTCGCTATGGTTCGTC-3′; CD40 reverse, 5′-AAGGCATTCCGTTTCAGT-3′; CD80 forward, 5′-ACCATCCAAGTGTCCATACCTC-3′; CD80 reverse, 5′-CAGCACCATTTTCTTCTCCTTT-3′; β-actin forward, 5′-AAGTACTCCGTGTGGATCGG-3′; β-actin reverse, 5′-ATGCATTCACCTCCCCTGTG-3′. Genes above were quantified using DreamTaq™ Green PCR Master Mix. ABI 7500 Fast Real-Time PCR platform (Applied Biosystems, Thermo Fisher Scientific, Waltham, MA, USA) conditions were as follows: 94°C for 5 min; 94°C for 40 sec, 55°C for 40 sec and 72°C for 40 sec, for 34 cycles for MHC I and 36 cycles for all others, followed by 72°C for 7 min. The amplified products were separated on a 3% agarose gel and visualized after 5 *μ*g/ml ethidium bromide (Sigma-Aldrich) staining for 10 min.

### Western blot analysis

HL-60 cells from all five groups were lysed in ice-cold Laemmli lysis buffer (cat. no. 38733; Sigma-Aldrich). The protein concentrations were measured using the coomassie brilliant blue method (Brilliant Blue G-250; Sigma-Aldrich) (22). Protein samples were separated using SDS-PAGE and then transferred to nitrocellulose membranes at a voltage of 100 V for 100 min. Following staining with Ponceau S solution, samples were blocked with 5% skimmed milk in PBS with Tween 20 at room temperature for 2 h. The membranes were incubated with rabbit anti-human CIITA (1:1,000) and β-actin (1:1,000) primary antibodies at 4°C overnight, and subsequently with a horseradish peroxidase-conjugated polyclonal goat anti-rabbit secondary antibody (1:3,000; cat. no. A24537; Invitrogen Life Technologies) at room temperature for 3 h. Blots were visualized using an enhanced chemiluminescence reagent (Invitrogen) and a LAS-3000mini luminoimage analyzer (Fujifilm, Tokyo, Japan).

### DNA bisulfite treatment

Genomic DNA of HL-60 cells was isolated with proteinase K (0.5%)/SDS (20 mg/ml) and identified using a UV spectrophotometer (Shimadzu UV2550) as previously described (23). The DNA was then treated with sodium bisulfite using the EZ DNA methylation-Direct kit according to the manufacturer’s instructions. Briefly, 500 ng DNA was denatured for 10 min at 9°C and incubated for 2.5 h at 64°C in 130 *μ*l CT Conversion Reagent. Subsequently, 600 *μ*l M-Binding Buffer was added and the mixture was centrifuged (12,000 × g) for 2 min prior to the addition of 200 *μ*l M-Wash Buffer. Following centrifugation, the samples were incubated with 200 *μ*l M-Desulphonation Buffer for 15-20 min at room temperature and washed with M-Wash Buffer twice. Finally, 10 *μ*l M-Elution Buffer was added and the DNA precipitate was eluted by centrifugation (12,000 × g, 4 min).

### Bisulfite-sequencing PCR (BSP) analysis

**Figure 1:**
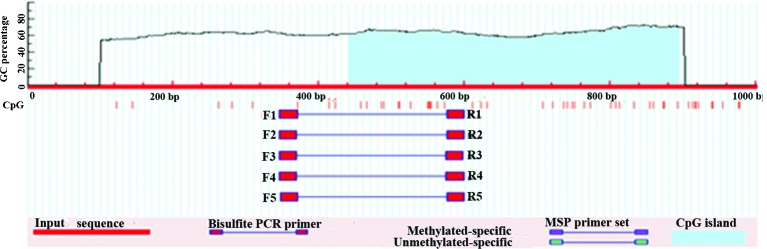
CpG sites located on the transcription start site in proximity to CIITApIV. Criteria used: Island size, >200; GC percentage, >50.0%; observed/expected, >0.6. CpG island 1: Start, 441; end, 895; size, 455 bp; observed/expected, 0.65; GC percentage, 55%. CIITApIV, class II transactivator promoter IV; CpG, cytosine-phosphate-guanine; PCR, polymerase chain reaction; MSP, monosulfite PCR.

Using CpG Island Searcher (http://cpgislands.usc.edu/), a DNA sequence of ~1,000 bp was analyzed, which was located on the transcription start site of CIITapIV. A CpG island comprising >200 bp (observed CpGs/expected CpGs>0.6, GC>50%) was selected (Fig. 1). Primers (Sangon Biotech Co., Ltd.) were as follows: Forward, 5′-TTGGGATGTTATTTTTGATAAAGTA-3′ and reverse, 5′-ACAAAAAAAACTTTAATCACCTACC-3′. Using DreamTaq™ Green PCR Master Mix, PCR (ABI 7500 Fast Real-Time PCR platform) was performed in a volume of 20 *μ*l containing 2 *μ*l buffer (10X), 0.5 *μ*l deoxynucleotide triphosphates (10 mM), 0.5 *μ*l Taq enzyme, 0.5 *μ*l Primer F (10 mM), 0.5 *μ*l of Primer R (10 mM), 14 *μ*l ddH_2_O and 2 *μ*l DNA template. Reaction conditions were as follows: 94°C for 3 min; 94°C for 30 sec, 53°C 30 sec, 72°C for 40 sec for 35 cycles, followed by 72°C for 5 min. The amplified products were separated on a 3% agarose gel and visualized after ethidium bromide (Sigma-Aldrich) staining.

### Cloning and sequence analysis

To sequence the bisulfite-PCR products, amplified fragments were spliced into pMD18-T vector using pMD18-T Vector Cloning kit from Takara Company. The cloning was performed in a volume of 10 *μ*l containing 1 *μ*l pMD18-T vector, 1 *μ*l PCR product, 3 *μ*l dH_2_O and 5 *μ*l Solution I at 16°C for 60-120 min. Following mixing with 100 *μ*l DH5a competent cells, the mixture above was put on ice for 30 min, followed by heating at 42°C for 60 sec. Finally, the mixture was cultured with agitation in 890 *μ*l super optimal broth medium at 37°C for 8 h, after which individual bacterial colonies were formed in LB-agar medium containing LB-agar medium containing 20mg/ml X-gal, 24 mg/ml IPTG and 100 mg/ml ampicillin (24). The next day, the growth of bacterial colonies was observed, and positive clones as blue or white plaques were screened. The selected clones were inoculated with agitation at 37°C overnight in 5 ml Luria-Bertani medium containing ampicillin (1:1,000). Universal primers of recombinant plasmids were as follows: Forward, 5′-GAGCGGATAACAATTTCACACAGG-3′ and reverse, 5′-CGCCAGGGTTTTCCCAGTCACGAC-3′. Recombinant plasmid was detected by PCR. Five positive clones were selected randomly to be sequenced from each recombinant colony. The DNA was sequenced using an ABI 3100 automated sequencer (Applied Biosystems) and gene sequence alignment was performed using DNASTAR-Lasergene v6 software (DNASTAR, Inc., Madison, WI, USA).

### Statistical analysis

All values are expressed as the mean ± standard error of the mean and analyzed using SPSS 10.0 software (SPSS, Inc., Chicago, IL, USA). The *t*-test was used for comparison of two groups, while single factor analysis of variance was used for comparison of multiple groups. P<0.05 was considered to represent a significant difference.

## Results

### Effect of epigenetic modification on expression of MHC molecules, CD40 and CD80

Using RT-PCR, the expression of MHC molecules as well as CD40 and CD80 was examined in HL-60 cells. mRNA levels of MHC class I, MHC class II, CD40 and CD80 were calculated as the integrated optical density (IOD) ratio to β-actin. The results showed that mRNA levels of MHCI, CD40 and CD80 were all significantly increased in the three epigenetic modification groups compared with those in the IFN-γ and control groups (P<0.05) (Table I).

**Figure 2:**
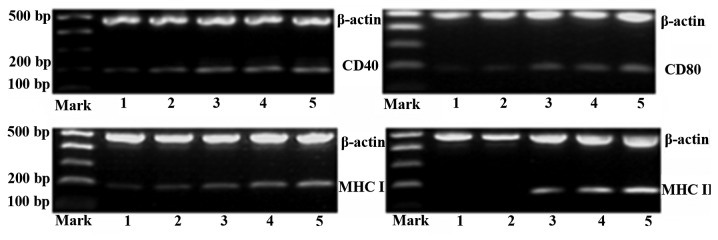
Electrophoretic analysis of the expression of MHC Class I and II as well as CD40 and CD80 in HL-60 cells. Lanes 1-5 represent groups A-E, respectively. Groups: A, phosphate-buffered saline; B, IFN-γ; C, 5-Aza-CdR (0.1 *μ*M) + SAHA (0.5 *μ*M) + IFN-γ; D, 5-Aza-CdR (1 *μ*M) + SAHA (0.5 *μ*M) + IFN-γ; E, 5-Aza-CdR (10 *μ*M) + SAHA (0.5 *μ*M) + IFN-γ. MHC, major histocompatibility complex; IFN, interferon; SAHA, suberoylanilide hydroxamic acid; 5-Aza, 5-aza-2′-deoxycytidine.

The expression of MHC class II gene was not detectable in the control and IFN-γ groups (Fig. 2 and Table I). However, following treatment with 5-Aza-CdR + SAHA + IFN-γ, HL-60 cells re-expressed MHC class II (0.146±0.011 in group C, 0.314±0.011 in group D and 0.368±0.019 in group E), and the expression of MHC class II was increased by 5-Aza-CdR in a concentration-dependent manner (P<0.05) (Table I).

### Effect of epigenetic modification on the expression of CIITA protein

**Figure 3:**
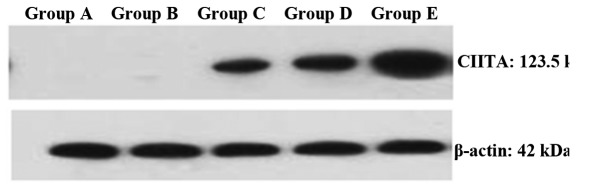
Expression of CIITA protein in HL-60 cells. Groups: A, phosphate-buffered saline; B, IFN-γ; C, 5-Aza-CdR (0.1 *μ*M) + SAHA (0.5 *μ*M) + IFN-γ; D, 5-Aza-CdR (1 *μ*M) + SAHA (0.5 *μ*M) + IFN-γ; E, 5-Aza-CdR (10 *μ*M) + SAHA (0.5 *μ*M) + IFN-γ. IFN, interferon; SAHA, suberoylanilide hydroxamic acid; 5-Aza-CdR, 5-aza-2′-deoxycytidine; CIITA, class II transactivator.

CIITA protein was not detectable in the control and IFN-γ groups (Fig. 3). However, the expression of CIITA protein increased dramatically following epigenetic modification, and the expression was significantly higher in group E compared with that in the other two epigenetic modification groups. This 5-Aza-CdR concentration-dependent increase in CIITA expression was in parallel to that of MHC class II, which implies that there may be a link between MHC class II and CIITA.

### PCR identification of CIITApIV gene and recombinant PMD18-T vector

**Figure 4:**
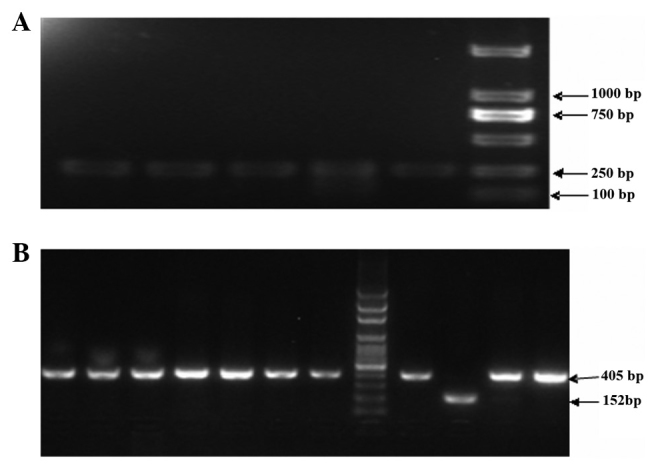
Electrophoretic analysis of PCR products. (A), BSP amplification products of CIITApIV; (B), bacterial colony PCR of positive recombinant plasmids. PCR, polymerase chain reaction; BSP, bisulfite-sequencing PCR; CIITApIV, class II transactivator promoter IV.

Following treatment with bisulfite, total DNA of leukemia cells was analyzed by BSP, and amplified BSP products were electrophoresed on 1.5% agarose gel. The length of BSP product was 253 bp, which was the expected length of the amplified fragment (Fig. 4A). Using colony PCR, it the correctness of the recombinant plasmids was further confirmed. The results showed that the amplified 152-bp fragment was the PMD18-T vector, while the 405-bp fragment was the recombinant plasmid, which had been inserted into the target gene (Fig. 4B).

### Effect of epigenetic modification on CpG island methylation of CIITA-pIV

**Figure 5:**
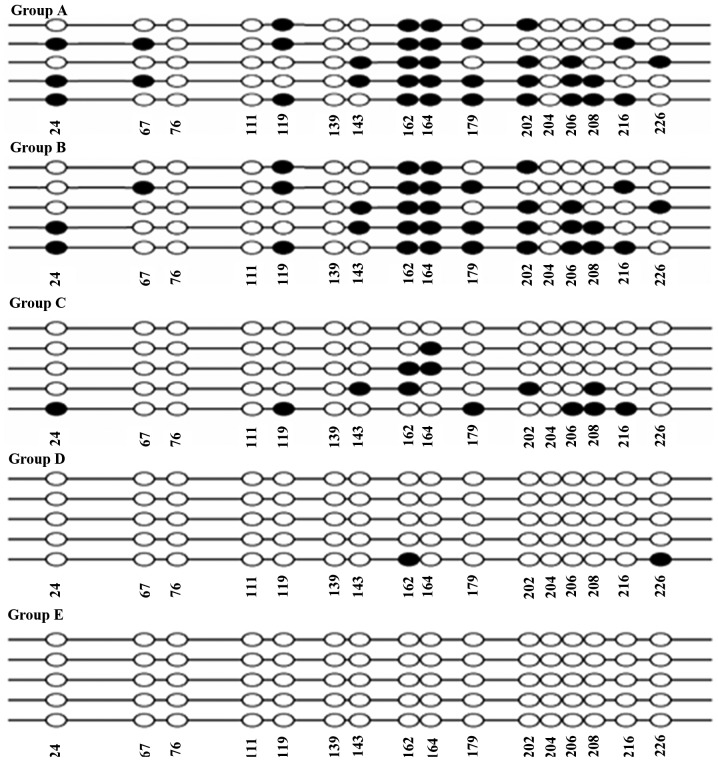
Bisulfite-sequencing of CIITApIV in bacterial colonies of different groups. Sites in black represent DNA hypermethylation, while white represents normal non-methylated sites. Groups: A, phosphate-buffered saline; B, IFN-γ; C, 5-Aza-CdR (0.1 *μ*M) + SAHA (0.5 *μ*M) + IFN-γ; D, 5-Aza-CdR (1 *μ*M) + SAHA (0.5 *μ*M) + IFN-γ; E, 5-Aza-CdR (10 *μ*M) + SAHA (0.5 *μ*M) + IFN-γ. IFN, interferon; SAHA, suberoylanilide hydroxamic acid; 5-Aza-CdR, 5-aza-2′-deoxycytidine; CIITApIV, class II transactivator promoter IV.

DNA sequencing results of CIITApIV showed that there were 16 CpG island sites, which were able to be methylated. Under the premise of at least five clones being sequenced for each group, it was found that methylation of the 162-, 164-, 179-, 202- and 206-bp sites occurred more frequently and the 162- and 164-bp sites of all five clones were methylated in groups A and B. The methylation rates of groups A and B (35/80 for group A and 33/80 for group B) were significantly higher than those in the the three epigenetics modification groups (P<0.05) (Table II and Fig. 5). The methylation rate decreased with increasing of 5-Aza-CdR concentration, and when the concentration of 5-Aza-CdR increased to 10 *μ*M, CIITApIV was completely demethylated (13/80 for group C, 6/80 for group D and 0 for group E) (Fig. 5 and Table II).

## Discussion

Antigen-specific T cells are a major force to induce anti-tumor immune response, and its activation depends on a dual signal (25). Following antigen presentation by MHC molecules, tumor antigens are recognized by the T-cell receptor (TCR) and hence the first signal for T-cell activation is transmitted (26). The transmission of the second signal depends on the mutual recognition between tumor cells and T-cell co-stimulatory molecules (27). If the number of first signals is not sufficient or if the second signal is absent, T cells are disabled (28). Antigen presentation by MHC class I molecules can activate CD8+ T cells, which is the main anti-tumor immune effector in cells (29). However, thorough activation of cytotoxic T-lymphocytes (CTL), the participation of CD4+ T cells is also required, whose receptor (CTL) recognizes the presenting antigens via MHC class II (30). Thus, once tumor antigens are not presented effectively by MHC molecules, antigen-specific T cells cannot be activated, and consequently, tumor cells evade being attacked by the immune system.

In a previous study, following treatment with the HDAC-1-specific inhibitor MS-275, the expression of CIITA and MHC class II in diffuse large B-cell lymphoma (DLBCL) cells was upregulated (21). Furthermore, the addition of HDACi trichostatin A (TSA) enhanced the expression of MHC class I and II, as well as the co-stimulatory molecule CD40 on the human neuroblastoma tumor cell line SK-N-MC (31). The MHC surface expression on tumor cells not only enhanced the anti-tumor immune response but also reduced tumorigenicity (32,33). There is currently no research regarding whether leukemia cells evade immune responses through reduced expression of MHC and co-stimulatory molecules. Therefore, the present study determined the expression of MHC molecules on the leukemia cell line HL-60 and found that the expression of MHC class II was very low at undetectable levels, even following stimulation with IFN-γ. This indicated that tumor antigen-specific CD4+ T cells cannot be effectively activated following contact with leukemia cells. Furthermore, activation of CTL and antibody production were affected (30). However, when mouse tumor-infiltrated CD11b myeloid cells were treated with DNMTi 5-Aza-CdR, cells were able to differentiate into mature antigen-presenting cells (34). Here, when HL-60 cells were pre-treated with 5-Aza-CdR + SAHA followed by IFN-γ stimulation, the expression of MHC class I, CD40+ and CD80+ significantly increased and expression of MHC class II genes was restored. This showed that the effect of 5-Aza-CdR and SAHA on HL-60 cells is non-specific. By elevating the expression of MHC class I, II and co-stimulatory molecules in leukemia cells, they may be transformed into antigen-presenting cells *in vivo*, which may be employed as an efficient anti-leukemia therapy. In this way, the proliferation and activation of CD4+ T cells and CD8+ T cells may be enhanced, which then activates the anti-tumor immune response. This may provide novel ways for the immunotherapy of leukemia.

At the same time, the demonstrated feasibility of restoring the expression of MHC class II by the epigenetic modification of 5-Aza-CdR + SAHA + IFN-γ on HL-60 cells suggested that there may be a direct association between absence of MHC class II and epigenetic abnormalities. A previous studies has shown that DNA hypermethylation and histone deacetylation in tumor cells may inhibit not only the expression of MHC II, but also certain co-stimulatory molecules and tumor-associated antigens (35). As a molecular switch of MHC II, CIITA may quantitatively control the expression of MHC II in a variety of cells (36,37). Therefore, the present study assessed the effect of changes in the methylation status of CIITA on the expression of class II MHC molecules in HL-60 cells.

In a variety of tumor cells, stimulation with IFN-γ increases the expression of MHC II through the activation of CIITApIV (12), while hypermethylation and deacetylation were shown to block the inductive effects of IFN-γ on CIITA in promyelocytic cells and breast cancer cells (38,39), which thereby enabled tumor cells to evade immune surveillance. Therefore, the inhibition of inductive effects of IFN-γ is closely associated with hypermethylation of CIITApIV. Previous studies showed that epigenetic modifications contribute to transcriptional silencing of CIITA in human tumor cells (38,40). However, this effect can be reversed by inhibitors of epigen-etic modifications. The HDACi TSA restored the expression of CIITA in rhabdomyosarcoma RD cells, and co-treatement of a DNMTi and TSA restored CIITA expression in SJRH30 cells (20). Simultaneous treatment of IFN-γ and TSA activated CIITA transcription in mouse trophoblasts (41). Previous studies also found that the hypermethylated tumor-suppressor genes MlH1, TIMP3, p15 and p16 were not able to be activated by TSA alone in tumour cells; however, when a low dose of 5-Aza-CdR for slight demethylation was added, TSA treatment resulted in the restoration of the expression of all genes stated above (42). Furthermore, the combination of DNA methylation inhibitor 5-Aza-CdR with histone deacetylase inhibitor TSA or FR901228 produced a greater inhibition of growth and DNA synthesis and a greater loss of clonogenicity than either agent alone in myeloid leukemic cells (43). In conclusion, DNA demethylation and HDAC inhibition have a synergistic effect on the restoration of the expression of antioncogenes, which had been de-activated by methylation or acetylation. Cells were treated with 5-Aza-CdR, SAHA and IFN-γ cooperatively for the reversal of epigenetic modification in the present study, leading to efficient restoration of the expression of molecules required for immune recognition.

The present study showed that the CIITA protein on leukemia HL-60 cells was not detected following stimulation with IFN-γ. Furthermore, hypermethylation of CpG islands in the CIITApIV gene promoter was not significantly changed following IFN-γ treatment. However, following deacetylation and demethylation with 5-Aza-CdR + SAHA prior to stimulation with IFN-γ significantly decreased CpG island methylation, and CIITA and expression was restored in parallel with that of MHC class II. This indicated that the loss in expression of MHC II was caused by the absence of CIITA, which was epigenetically regulated by CpG island hypermethylation of the CIITApIV promoter in leukemia HL-60 cells. The results of the present study indicated that treatment with 5-Aza-CdR + SAHA + IFN-γ may be an efficient strategy to restore the immune recognition of leukemia cells as a treatment strategy.

## Figures and Tables

**Table I:** mRNA levels of MHC class I, II, CD40 and CD80.

IOD ratio	Group A	Group B	Group C	Group D	Group E
MHC-I/β-actin	0.036±0.011	0.046±0.012	0.092±0.008	0.146±0.010	0.218±0.022
MHC-II/β-actin	0	0	0.146±0.011	0.314±0.011	0.368±0.019
CD40/β-actin	0.058±0.016	0.060±0.014	0.170±0.019	0.202±0.017	0.258±0.021
CD80/β-actin	0.052±0.008	0.058±0.009	0.112±0.015	0.160±0.016	0.178±0.013

Values are expressed as the mean ± standard error of the mean. Groups: A, phosphate-buffered saline; B, IFN-γ; C, 5-Aza-CdR (0.1 *μ*M) + SAHA (0.5 *μ*M) + IFN-γ; D, 5-Aza-CdR (1 *μ*M) + SAHA (0.5 *μ*M) + IFN-γ; E, 5-Aza-CdR (10 *μ*M) + SAHA (0.5 *μ*M) + IFN-γ. MHC, major histocompatibility complex; IOD, integrated optical density; IFN, interferon; SAHA, suberoylanilide hydroxamic acid; 5-Aza-CdR, 5-aza-2′-deoxycytidine.

**Table II:** Effect of epigenetics modification on CpG island methylation of CIITApIV.

Group	CpG methylation rate
A	35/80^a^
B	33/80^a^
C	13/80
D	6/80
E	0/80

Groups: A, phosphate-buffered saline; B, IFN-γ; C, 5-Aza (0.1 *μ*M) + SAHA (0.5 *μ*M) + IFN-γ; D, 5-Aza (1 *μ*M) + SAHA (0.5 *μ*M) + IFN-γ; E, 5-Aza (10 *μ*M) + SAHA (0 .5 *μ*M) + IFN-γ. The methylation rate was calculated as the ratio of the number of methylated clones to the number of total clones;

a P<0.05, vs. groups C, D and E. MHC, major histocompatibility complex; IFN, interferon; SAHA, suberoylanilide hydroxamic acid; 5-Aza-CdR, 5-aza-2′-deoxycytidine; CIITApIV, class II transactivator promoter IV; CpG, cytosine-phosphate-guanine.

## References

[1] McKenna SJ (2000). Leukemia. Oral Surg Oral Med Oral Pathol Oral Radiol Endod.

[2] Klippel ZK, Chou J, Towlerton AM (2014). Immune escape from NY-ESO-1-specific T-cell therapy via loss of heterozygosity in the MHC. Gene Ther.

[3] Siddle HV, Kreiss A, Tovar C (2013). Reversible epigenetic down-regulation of MHC molecules by devil facial tumour disease illustrates immune escape by a contagious cancer. Proc Natl Acad Sci USA.

[4] Wolkersdörfer T, Fussel M, Kiesslich T (2011). MHC class II genotype- and MHC class I and II phenotype-related parameters in sporadic colorectal cancer. Oncol Rep.

[5] Xu WC, Li ZB, Chen YR (2011). Expression and distribution of S-100, CD83 and costimulatory molecules (CD80 and CD86) in tissues of thyroid papillary carcinoma. Cancer Invest.

[6] Fernando MM, Stevens CR, Walsh EC (2008). Defining the role of the MHC in autoimmunity: A review and pooled analysis. PLoS Genet.

[7] Choi NM, Majumder P, Boss JM (2011). Regulation of major histocompatibility complex class II genes. Curr Opin Immunol.

[8] Devaiah BN, Singer DS (2013). CIITA and its dual roles in MHC gene transcription. Front Immunol.

[9] Otten LA, Steimle V, Bontron S, Mach B (1998). Quantitative control of MHC class II expression by the transactivator CIITA. Eur J Immunol.

[10] Green MR, Yoon H, Boss JM (2006). Epigenetic regulation during B cell differentiation controls CIITA promoter accessibility. J Immunol.

[11] Chen H, Gilbert CA, Hudson JA, Bolick SC, Wright KL, Piskurich JF (2007). Positive regulatory domain I-binding factor 1 mediates repression of the MHC class II transactivator (CIITA) type IV promoter. Mol Immunol.

[12] Pisapia L, Pozzo GD, Barba P, Citro A, Harris PE, Maffei A (2012). Contrasting effects of IFNalpha on MHC class II expression in professional vs. nonprofessional APCs: Role of CIITA type IV promoter. Results Immunol.

[13] Baylin SB, Jones PA (2011). A decade of exploring the cancer epigenome-biological and translational implications. Nat Rev Cancer.

[14] Sandoval J, Esteller M (2012). Cancer epigenomics: beyond genomics. Curr Opin Genet Dev.

[15] Burke MJ, Bhatla T (2014). Epigenetic modifications in pediatric acute lymphoblastic leukemia. Front Pediatr.

[16] Chatterton Z, Burke D, Emslie KR (2014). Validation of DNA methylation biomarkers for diagnosis of acute lymphoblastic leukemia. Clin Chem.

[17] Nordlund J, Backlin CL, Wahlberg P (2013). Genome-wide signatures of differential DNA methylation in pediatric acute lymphoblastic leukemia. Genome Biol.

[18] Savickiene J, Treigyte G, Valiuliene G, Stirblyte I, Navakauskiene R (2014). Epigenetic and molecular mechanisms underlying the antileukemic activity of the histone deacetylase inhibitor belinostat in human acute promyelocytic leukemia cells. Anticancer Drugs.

[19] Xu WS, Parmigiani RB, Marks PA (2007). Histone deacetylase inhibitors: molecular mechanisms of action. Oncogene.

[20] Londhe P, Zhu B, Abraham J, Keller C, Davie J (2012). CIITA is silenced by epigenetic mechanisms that prevent the recruitment of transactivating factors in rhabdomyosarcoma cells. Int J Cancer.

[21] Cycon KA, Mulvaney K, Rimsza LM, Persky D, Murphy SP (2013). Histone deacetylase inhibitors activate CIITA and MHC class II antigen expression in diffuse large B-cell lymphoma. Immunology.

[22] Candiano G, Bruschi M, Musante L (2004). Blue silver: A very sensitive colloidal Coomassie G-250 staining for proteome analysis. Electrophoresis.

[23] Dodd KW, Burns TC, Wiesner SM (2011). Transgenic mice expressing luciferase under a 4.5 kb tyrosine hydroxylase promoter. Cureus.

[24] Baev MV, Baev D, Radek AJ, Campbell JW (2006). Growth of Escherichia coli MG1655 on LB medium: Monitoring utilization of sugars, alcohols, and organic acids with transcriptional micro-arrays. Appl Microbilol Biot.

[25] Cohn M (2009). How does the immune response get started?. Cell Immunol.

[26] Weiss A, Imboden J, Hardy K (1986). The role of the T3/antigen receptor complex in T-cell activation. Annu Rev Immunol.

[27] Santos DO, Miranda A, Suffys P (2007). Current understanding of the dendritic cells and their co-stimulatory molecules as a key in generating efficient T cell responses in lepromatous leprosy. Curr Immunol Rev.

[28] Shafer-Weaver K, Anderson M, Malyguine A, Hurwitz AA (2007). T cell tolerance to tumors and cancer immunotherapy. Adv Exp Med Biol.

[29] Ueki T, Murata S, Kitamura N, Mekata E, Tani T (2009). Pre-treatment with cyclophosphamide or OX40 (CD134) costimulation targeting regulatory T cell function enhances the anti-tumor immune effect of adoptively transferred CD8+ T cells from wild-type mice. Mol Med Rep.

[30] Hung K, Hayashi R, Lafond-Walker A (1998). The central role of CD4(+) T cells in the antitumor immune response. J Exp Med.

[31] Magner WJ, Kazim AL, Stewart C (2000). Activation of MHC class I, II and CD40 gene expression by histone deacetylase inhibitors. J Immunol.

[32] Garrido C, Paco L, Romero I (2012). MHC class I molecules act as tumor suppressor genes regulating the cell cycle gene expression, invasion and intrinsic tumorigenicity of melanoma cells. Carcinogenesis.

[33] Seliger B (2014). The link between MHC class I abnormalities of tumors, oncogenes, tumor suppressor genes and transcription factors. J Immunotoxicol.

[34] Daurkin I, Eruslanov E, Vieweg J, Kusmartsev S (2010). Generation of antigen-presenting cells from tumor-infiltrated CD11b myeloid cells with DNA demethylating agent 5-aza-2′-deoxycytidine. Cancer Immunol Immunother.

[35] Sigalotti L, Coral S, Fratta E (2005). Epigenetic modulation of solid tumors as a novel approach for cancer immunotherapy. Semin Oncol.

[36] Reith W, Muhlethaler-Mottet A, Masternak K, Villard J, Mach B (1999). The molecular basis of MHC class II deficiency and transcriptional control of MHC class II gene expression. Microbes Infect.

[37] Ulbricht T, Alzrigat M, Horch A (2012). PMl promotes MHC class II gene expression by stabilizing the class II transactivator. J Cell Biol.

[38] Truax AD, Thakkar M, Greer SF (2012). Dysregulated recruitment of the histone methyltransferase EZH2 to the class II transactivator (CIITA) promoter IV in breast cancer cells. PLoS One.

[39] De Lerma Barbaro A, De Ambrosis A, Banelli B (2008). Methylation of CIITA promoter IV causes loss of HLA-II inducibility by IFN-gamma in promyelocytic cells. Int Immunol.

[40] Radosevich M, Song Z, Gorga JC, Ksander B, Ono SJ (2004). Epigenetic silencing of the CIITA gene and posttranscriptional regulation of class II MHC genes in ocular melanoma cells. Invest Ophthalmol Vis Sci.

[41] Holtz R, Choi JC, Petroff MG, Piskurich JF, Murphy SP (2003). Class II transactivator (CIITA) promoter methylation does not correlate with silencing of CIITA transcription in trophoblasts. Biol Reprod.

[42] Cameron EE, Bachman KE, Myohanen S, Herman JG, Baylin SB (1999). Synergy of demethylation and histone deacetylase inhibition in the re-expression of genes silenced in cancer. Nat Genet.

[43] Shaker S, Bernstein M, Momparler LF, Momparler RL (2003). Preclinical evaluation of antineoplastic activity of inhibitors of DNA methylation (5-aza-2′-deoxycytidine) and histone deacetylation (trichostatin A, depsipeptide) in combination against myeloid leukemic cells. Leuk Res.

